# A Plea for the Antiphlogistic Treatment of Disease

**Published:** 1872-08

**Authors:** Edward Montgomery

**Affiliations:** St. Louis


*A Plea for the Antiphlogistic Treatment of Disease.* By EDWARD MONTGOMERY, M.D., St. Louis. Read before the Medical Association of the State of Missouri.

In presenting a few thoughts on the antiphlogistic treatment of disease, I would explain that I use the term, "Antiphlogistic," because of its comprehensiveness, and the ideas it is intended to convey being so well and so universally understood, and not from its etymology or the doctrines and theories, now perhaps obsolete, which it was formerly believed to denote. I also wish it to be distinctly understood that I do not hold bloodletting to be the chief, principal and main remedy in the antiphlogistic treatment; but that I also recognize purgatives, mercurials, arterial and nervous sedatives, anodynes, anti-zymotics, anti-toxæmics, refrigerants, diuretics and diaphoretics, and an appropriate dietetic and general hygienic regimen, as all going to form this system of therapeutics.

There is no doubt but that a great amount of the obloquy and disrespect cast upon this system of medication arises from the false ideas and wrong constructions put upon it. It has been construed to imply bleeding, *ad deliquum*, mercurialization to salivation, purgation to hypercatharsis, etc.; and this depleting and debilitating system to be kept up to the end of the case, with little regard to stage or type of the malady, or the age, idiosyncrasy or constitution of the patient. Hippocrates, Galen, Areteus, Oribasius, Alexander of Trulles, Paulus Aegenita, Cælius Aurelianus, John Fernel, Sydenham, Cullen, Bœrhaave, Gregory, Andral, Rush, Physick, the Hunters, Mead, Marshall Hall, Trousseau, Dunglison, Watson, Atkin, Copland, Tweedie, Williams, and, in short, all the principal authors of ancient and modern times, have advocated the antiphlogistic treatment of inflammatory diseases; and yet we are to set no value, attach no importance to the experience of the past, or the clinical knowledge of our learned predecessors. It is rather suggestive that the antiphlogistic treatment was ridiculed and opposed by the arrogant mountebanks who persecuted Galen during his residence at Rome; and during the dark ages it was almost forgotten, together with the learning of the Greek sages, and instead there was substituted the alexipharmics and superstitious forms and manipulations of the Arabian doctors. Early in the sixteenth century bloodletting was again brought into notice by Pierre Brissot, of Paris, who published a treatise under the name of "An Apology for the Employment of Bloodletting in Inflammatory Diseases, especially Pleuritis." It appears there was a severe epidemic in France at that time, attended with great fatality, which was supposed to be a pleurisy or pleuro-pneumonia—most likely the latter, from the mortality attending it, and Dr. Brissot, sadly contemplating the unsuccessful treatment then in vogue, resolved to try the long-neglected and almost forgotten system of depletion practiced and recommended by Hippocrates, Galen and Areteus, and, meeting with wonderful success, he wrote the treatise advocating the copious abstraction of blood. Although the revival of this system met with stern and systematic opposition, it was, doubtless, Pierre Brissot to whom the credit belongs of again popularizing the system in Europe in the year 1525. In the year 1601 was born Guy Patin, a very learned and accomplished physician of Paris, who turned out to be one of the most daring and boldest phlebotomists the world ever produced. He boasted of curing his own son of a continued fever "by twenty-three bleedings from the arms and feet, with at least a dozen good purgatives of cassia, senna and syrup of pale roses." . . . . "About the year 1633, M. Condinot, now first physician to the king, was attacked with a severe and violent rheumatism, for which I bled him sixty-four times, after which I purged him, and he was completely cured." He also relates the case of a boy, aged seven years, whom he bled thirteen times and cured of a pleurisy.

About the middle of the sixteenth century, Leonard Botullus also boldly advocated free venesection in a great variety of diseases, and among all classes of patients. He carried the idea so far as to advise that an infirm old man should be bled from four to six times a year, and that it was a good custom to open a vein every six weeks in young and healthy individuals. Although the Paris Faculty very properly condemned his practice as dangerous and heretical, he was said to have met with great success, and to have cured a great number of patients.

Dr. Gregory, of Edinburgh, used to relate to his students the case of a young girl who was bled to one hundred and thirty ounces within a few days before her disease (pleurisy) was subdued; and also the case of Dr. Radcliff, who in one day was bled to the extent of one hundred ounces, and adds that he (Dr. Radcliff) at least was no fool; for had he not felt assured that the depletion was necessary for the cure of his disease he would not have permitted it.

Dr. Boulland advised bleeding *coup sur coup* in pneumonia, and determined the medium quantity to be taken in three days at from ninety to one hundred and twenty ounces. Of one hundred and two cases treated in this way, ninety were cured and twelve died; average duration of the disease, nine to thirteen days. It is stated in Dr. Samuel Cooper's work on surgery, that in surgical annals many cases are given where patients were rescued from death by means of bloodletting to the amount of two or three hundred ounces within the space of two or three days. Dr. Watson relates in his lectures the case of a robust young man whom he saw bled at once to the extent of seventy-two ounces for the cure of general dropsy of recent date. This occurred in the Edinburgh Royal Infirmary, and the patient not only survived the heroic treatment, but was promptly cured. Dr. Tweedie, in the *Encyclopædia of Practical Medicine*, says that he bled a patient suffering from pericarditis, to the amount of sixty ounces at once, with decided relief; and yet our modern medical reformers totally condemn all the antiphlogistic remedies in this disease. Dr. Rush, of Philadelphia, speaks of bleeding a four-year-old boy four times in one day, and quotes Dr. Physick approvingly for bleeding a child aged three months three times in one day and giving it half a drachm of calomel in the same space of time. The child had croup, and not only resisted this barbarous treatment, but actually got well. We all know of the enormous quantities of calomel, tartar emetic and lobelia which were administered by our own physicians thirty years ago; but whilst condemning and repudiating this exhausting and debilitating treatment, we should learn from it that abstracting a few ounces of blood, or giving a few doses of calomel, tartar emetic, or veratrum viride, is not fraught with such direful consequences as Homœopathists and Expectants would have us believe. If patients not only survived, but actually made good recoveries after such extravagant use of the lancet, nauseants and purgatives, the antiphlogistic treatment, judiciously employed, cannot be such a dangerous system as represented by Tod, Skoda, Bennett, Tanner, etc. All engaged in extensive practice will have observed the frequent extensive hæmorrhages and great loss of blood sustained by paturient women, by parties wounded, and by those attacked by severe epistaxis, hæmatemesis, etc., and their rapid and complete recovery from these severe depletions. These facts go to prove that bloodletting is not the dangerous remedy some writers would lead us to believe; and this will be still more apparent when we remember that when we employ bleeding as a remedial measure, it is always in those cases of acute sthenic inflammations which bear depletion with such wonderful impunity. The same holds good with regard to the other antiphlogistic remedies. We all know with what tolerance the sufferer with pneumonia will take free and frequent doses of tartar emetic, or veratrum viride; and to what an extent purgatives can be administered to patients with encephalitis, not only without producing debility, but, on the contrary, reanimating and reviving their wonted energies. We would appeal to the deliberate reflection and calm judgment of every experienced physician, and ask him how often he has witnessed cases of acute sthenic inflammation injured by the antiphlogistic treatment; and, on the other hand, how many times he has had reason to regret the omission or inadequate employment of these measures. We are firmly convinced that for the last twenty years this treatment has been greatly overlooked or neglected, to the manifest detriment of the patient. Many cases might have been shortened, and much suffering, and in many instances permanent injury, might have been avoided by a timely, efficient and judicious application of this system of therapeutics.

There is a great tendency in the human mind to go into extremes; in medical science especially, opinions, theories and systems cannot maintain a middle course, but, like the vibrations of the pendulum, they must swing from one extreme to the other. There is no doubt but that venesection and most of the other antiphlogistic remedies were carried to great excess from the beginning of the sixteenth to the middle of the nineteenth centuries. From the writings of Pierre Brissot, Guy Patin, Leonard Botullus, John Fernel, and others, the depleting system of treatment was carried to great excess; but for the past twenty years the fashion has changed, and an extremely opposite system has extensively prevailed. Not only are venesections, mercurials, antimonials, purgatives and arterial sedatives almost entirely ignored, but a most demoralizing and unscientific system of stuffing and stimulating has been inaugurated and extensively practiced, the consequences of which have been lamentable and deplorable. Thousands of patients have been advised by their physicians to take a little brandy, gin, or rum, a glass of iced champagne, or pale sherry; or, if in the poorer ranks of life, an occasional dram of "old Bourbon," to keep up their strength and assist nature in throwing off the disease; and by this supporting treatment, as it is called, many are seduced into a state of chronic alcoholism, or at least a constant indulgence in the use of intoxicating beverages. This is one of the most deplorable results of the great rebound from the antiphlogistic to the so-called restorative or supporting system of medical practice. In almost every disease, whether pneumonia or typhoid fever, pericarditis or hysteria, whisky or wine, brandy or gin enters largely into the physician's prescriptions; a venesection or a blister, tartar emetic or calomel, would now be sneered at and condemned as antiquated, barbarous and vulgar, by the fashionable doctors of the present day; whilst alcoholic stimulation and high feeding are alike recommended for the patient laboring under an acute attack of pneumonitis, endocarditis, or typhoid fever. How potent is the influence of fashion, how strong the desire for everything that is novel and miraculous! If Cullen or Gregory, John Fernel or Benjamin Rush, could now witness the treatment of a pneumonia or a cerebritis with alcoholic slimulants and a most generous diet, the circumstance would seem to them most wonderful and inexplicable; but the ultra depletions and purgings of Guy Patin and Botullus have been superseded by the stimulating and stuffing of Tod and Bennett.

There is no doubt but that this great rebound from an excessive perturbating and debilitating to an excessive stimulating and overfeeding system, has partly resulted from the enormous extent to which the former system was carried during the past three centuries. As before intimated, there seems also to be an impression that the antiphlogistic treatment means an unvarying and uninterrupted course of bleeding, purging, nauseating and starving; whereas the scientific and judicious application of this system is very different from that. Hippocrates, Galen and Areteus gave very good rules for depletion and contra-stimulants; and it was only because the sound doctrines and clinical experience of the Greeks were forgotten in the dark ages that this system became obsolete. But in the erudite period, when Fernel and others revived the doctrines of the schools of Cos, of Pergamos, and of Alexandria, the depleting practice was carried to such excess by Brissot, Guy Patin, Botullus, and others, that even to this day this practice is confounded with a most devitalizing and perturbating one; so that a directly opposite course of supporting, stimulating and temporizing has very naturally occupied the minds of men so prone to swing from one extreme to the other. As first prominently set forth by Hippocrates, depleting remedies should only be given in the very first stages of the attack. In pneumonia, for instance, if venesection and veratrum viride are not employed in the very beginning of the disease they can do no good, but will certainly do great harm. If the plastic fibrinous exudation be already formed, bleeding and arterial sedatives are not indicated; but calomel, opium, tartar emetic, the iodide of potassium, etc., may, and we believe will, assist in producing resolution; or, if that is impossible, will circumscribe and modify the purulent infiltration. It should also be borne in mind that it is only in acute sthenic pneumonia in a vigorous subject that we would use venesection at all; in other cases, cups or leeches, followed by warm poultices or stimulating liniments, will take the place of large bleedings and extensive vesication. As soon as the high inflammatory symptoms are subdued, or whenever signs of prostration ensue, we believe there is no inconsistency or incompatibility in following our depleting measures with nutrients and tonics, such as quinine, carbonate of ammonia, tonic bitter infusions, cod-liver oil, beef essence, raw eggs, milk, etc.

The experiments of Beale, Sticker, Bilroth, Rindfleish, and especially of Cohnheim, make it evident that we as yet know of no remedy which will disperse or dissolve the migratory white blood corpuscles after they have passed through the coats of vessels, and become densely packed in the adjacent parenchymatous tissue; but before these changes take place we may, by a judicious selection and use of antiphlogistics, prevent, to a great extent, the force and pressure of the blood on the inflamed part, the dilatation of the vessels, which affords an easy exit to the migratory corpuscles, and consequently circumscribe and curtail the extent of the exudation. Although histologists do not positively assert that the migratory colorless blood corpuscles do constitute the whole pabulum of the inflammatory exudation, yet they do aver, "that the transition of the inflammation into suppuration is impelled by the unlimited continuance and superabundance of the formation, whereby in a short time a really colossal amount of young cells are furnished. The impulse to suppuration of the inflammatory infiltrate is also in many cases chiefly to be sought in the great intrusion of juices to the inflamed part, hence our antiphlogistic therapeutists always pre-eminently endeavor to prevent this intrusion of juices, or at least to diminish it." (Rindfleish, page 112.)

Now, Dr. Bennett's strongest argument against the antiphlogistic and in favor of the supporting treatment is, that we cannot curtail or diminish the exudation, but by stimulants and nutrients we can hasten the suppurative process and get rid of it in that way, which he says is the natural process. But we submit that we may to a great extent prevent the great intrusion of juices and lessen the colossal amount of young cells, as Rindfleish expresses it, and so circumscribe and limit the exudation that resolution may ensue, or even if suppuration takes place it will be to a more limited extent, and, of course, fraught with far less danger to the patient. In the commencement of any inflammatory disease, if the patient is not too young, too old, or too feeble for antiphlogistics, we bleed, give veratrum or aconite, and purge so as to limit the accumulation of these young cells and prevent the flow of juice to them, so as to circumscribe the inflammatory infiltrate and encourage its resolution, or, if that is impossible, limit the extent of purulent infiltration. After the stage of hepatization, as it is called, has been fully developed, we believe that the careful and judicious use of tartar emetic, calomel and opium will accomplish much good in assisting the natural processes to get rid of the fibrinous exudation.

Late physiological experiments seem to indicate that we may also do much to overcome the danger of the inflammatory exudation by giving cod-liver oil, which may hasten fatty degeneration, whereby it (the exudation) is turned into a "milky detritus," which can be absorbed. This reminds me of the fact that there is no scientific inconsistency in giving cod-liver oil and tartar emetic to the same patient at the same time; or in using quinine and venesection, or administering mercury and nutrients. We bleed, give purgatives and veratrum viride to lessen the amount of blood, prevent its local engorgement, quiet the action of the heart and arteries, allay irritation and give ease and repose to the general system, and immediately give quinine, cod-liver oil, bitter tonic decoctions and nutritive drinks to promote fatty degeneration of the cellular element of the inflammatory infiltrate, to limit or prevent purulent infiltration, to aid absorption, and to obviate the tendency to death by septicæmia, abscess, gangrene, or asthenia.

In order to illustrate what we mean by the antiphlogistic treatment of inflammatory diseases, we will give a brief outline of it in a few of the diseases in which this treatment has generally been adopted. At the commencement of a case of acute sthenic pneumonia in a robust subject, we would use venesection until approaching syncope, the patient sitting up; we would at once give a cathartic of calomel and jalap, unless the patient had been already purged; we would apply cloths wrung out of ice-water to the chest, and we would endeavor to keep the action of the heart and arteries quiet with aconite or veratrum viride. The venesection we hardly ever repeat, but might apply leeches or scarified cups. In the second stage we would keep the chest enveloped in a large flaxseed poultice, give small doses of tartar emetic, and two or three grains of calomel with one grain or half a grain of opium every three hours, taking particular care not to push the mercurial to salivation. In this stage a mixture of carbonate of potassa, syrup of senega and aqua laura cerasi will soothe the cough and counteract the viscid tenacity in the throat. Toward the advanced period of this stage cod-liver oil and quinine should also be freely administered, together with beef tea, soft boiled or raw eggs, cream, etc.

In the stage of purulent infiltration we would give cod-liver oil, quinine, vegetable tonic decoctions, carbonate of ammonia, and nutrients.

In the advanced stages of all cases we would give an occasional dose of some diuretic, as nitre and taxaxicum, fluid extract of cubebs, or of Pareira Brava. Blisters may now also be required and often do much good.

In cases of asthenic pneumonia, of course we would never resort to venesection; but in some instances cupping or leeching may be called for; opiates if much pain, cod-liver oil, quinine, tonic decoctions, carbonate of ammonia, with gum and infusion of wild cherry bark, and the large flaxseed poultice all around the chest; beef essence, eggs and cream.

In pleuritis, the packing with ice-cold cloths will be very apt to limit the inflammation; but if the patient is very plethoric and robust, a venesection will be of great service. Cups or leeches, followed by the large poultice, a small, frequently-repeated dose of tartar emetic, some calomel and opium, and in some cases fly-blisters.

In enteritis, if the patient is not too young, too old, or too feeble, the antiphlogistic treatment will also be appropriate; but here we must beware of purgatives; if we must give any, the oleaginous emulsion will be the most suitable and appropriate. Venesection, cupping or leeching, must only be resorted to if the case absolutely demands them, and they must only be employed at the very commencement of the disease. In this disease opiates are the sheet anchor; a combination of morphine and ipecacuanha is, we think, *the remedy* here; and there should be enough of the morphine administered to keep the patient perfectly calm and free from pain, even if it requires partial narcotism to do that. The constant application of warm emollient poultices to the abdomen will also be useful; very particular attention must be given to the diet and drinks allowed, both of which should be of a non-irritating and antiphlogistic character. In all inflammatory affections of the abdominal viscera, ipecacuanha, with opiates, will be found to have most happy effects—in acute dysentery, in hepatitis, enteritis and peritonitis, these medicines are invaluable. Dr. Hamilton, of Lynn Regis, Dr. Armstrong and Dr. Stokes all deserve credit for urging the claims of opiates in inflammatory disease. Dr. Stokes is quite enthusiastic in his praise, especially in enteritis and peritonitis; and to Dr. Alonzo Clark, of New York, the profession in this country are deeply indebted for so emphatically calling their attention to this remedy in *post partum* inflammations. As early as the year 1764 Dr. Hamilton wrote an article in praise of calomel and opium as an antiphlogistic remedy, especially in acute rheumatism, of which monograph Dr. Armstrong declared it should be written in letters of gold; and notwithstanding the efforts of those in favor of a homœopathic or an expectant method of treatment to cast discredit on this remedy, it is certainly one of the best and most efficient antiphlogistics we possess; of course, it is a combination which will very readily produce salivation, but this should be sedulously guarded against. In cerebritis, or, indeed, in any form of encephalitis, opiates should be used with great caution, and in a majority of these cases they are entirely inadmissible. Even venesection is rarely practiced in these cases; cups, leeches, blisters to neck, sinapisms to the spine and extremities, ice to the head, catharsis, with croton oil, aloes, gamboge, scammony, black hellebore, colocynth, calomel and jalap, or elaterium; and if the case is prolonged, iodide or bromide of potassium may be tried. In the epidemic cerebro spinal meningitis we have great faith in calomel and Dover's powder, sulphate of quinine and bromide of potassium. We first apply scarified cups to the back of the neck, sponge the head and neck with ice-water, give calomel and Dover's powder until the violent symptoms are subdued, then give large doses of quinine, alternating with full doses of the bromide of potassium. We cannot suppress the declaration, that those physicians who recommend an expectant or a supporting treatment to cerebritis or cerebral meningitis are seriously jeopardizing the lives of their patients; their treatment is but "a meditation on death." It is true that many cases of cerebritis and cerebral meningitis will have passed into effusion, softening, or even abscess, before we are aware, or before our treatment commences; but even then, cups, blisters, purgatives and iodide of potassium can do no harm; but the supporting treatment can do no good when the disease has progressed this far, and will do a great amount of harm if it has not. Suppose we are called to a patient with severe headache, intolerance of light and sound, the pulse accelerated and hard, the heat of skin above what is normal, nausea and perhaps vomiting, constipation, a peculiar feeling in one or more of the extremities, as if the nerves were ligated, and perhaps spasmodic twitchings or convulsions. Now, although we cannot say positively whether in this case we have only a hyperæmia, a cerebral congestion, an exudation, or ramollissement, it is plainly our duty to administer a purgative at once; and if the patient is young and robust we should also resort to venesection, or cup, or leech, and follow these measures with iodide of potassium, and apply ice to the head and sinapisms to the extremities; for if there is a clot already formed, or if there is softening or abscess, these prompt measures will do no harm; and if the case is only one of congestion or incipient inflammation, our active interference will likely arrest the disease, whereas if an expectant or a stimulating treatment is adopted, the disease may naturally progress or be aggravated into irremediable effusion, softening, or abscess. Whilst writing this, my attention was accidentally called to a case reported in the January number of the *London Lancet*, page 43, which vividly illustrates the happy effects of an antiphlogistic treatment in a case of great gravity. W. W., aged fifty, had been in bed all day, got up and went out at 7 P. M., when he fell down suddenly and was totally unconscious and could not be aroused. His pupils were dilated and insensible to light and touch, his breathing stertorous, the pulse hard and quick; he was bathed in perspiration, and the countenance was livid. . . . He was bled from the arm to 18 ounces without delay. As the blood was flowing there was a marked improvement in the symptoms; the breathing ceased to be stertorous, the countenance became less flushed, the pulse weaker; the patient, however, did not recover consciousness; but as matters progressed so well he was sent into the wards, where he was ordered to take a minim of croton oil, also five grains of the bromide of potassium every four hours, and to have ice applied to the head. He gradually recovered without any paralysis or any loss of power or sensibility of any of his limbs.

Drs. Bennett, Tanner, Flint, and others, who are such advocates of the supporting treatment, are continually harping on the great danger of the patient dying from debility, when the facts are that persons laboring under these inflammatory diseases, properly speaking, rarely ever die of asthenia. It would be about as correct to say that a man dying from a gunshot wound in the bowels, or a bayonet stab in the lungs, was dying from debility, as to designate the fatal termination of most of these cases of inflammation as "death by asthenia." If we fail to circumscribe or modify the inflammatory process, congestion, effusion, purulent infiltration or abscess will soon destroy life, and all the whisky and brandy in the universe will be perfectly powerless to sustain the organic and vital functions. In our overweening anxiety lest antiphlogistic measures should weaken and exhaust the patient, let us not forget that unrestrained and prolonged inflammation will soon accomplish that work, and without the least hope of reprieve or remedy.

But we deny that antiphlogistics judiciously employed in the proper stages of appropriate cases tend to produce death by asthenia; even in pneumonia, the typical disease so much depended upon by Todd, Bennett, Skoda, Dieth, etc., to prove the danger of antiphlogistic remedies, if a venesection is employed to relieve the pain, dyspnœa, pulmonary or cerebral congestion, or if veratrum viride is administered to lower the hard, rapid pulse and reduce the hyperpyrexia, will the patient be in any more danger of death by asthenia than if these remedies had not been employed? Certainly not, but the condition of the patient will be so much improved by them that in such conditions, instead of being depleting and debilitating measures, these means appear as restoratives; and let it be remembered that many remedies which we claim as acting as antiphlogistics in inflammatory diseases, are really restoratives and tonics. In many instances even venesection may act as an indirect tonic by promoting absorption, encouraging the action of nutrients and medicines, removing engorgement, congestion, etc. Calomel also often counteracts debility by promoting the secretions, improving nutrition, aiding resolution, etc. We presume no one will gainsay the tonic properties of cod-liver oil and quinine, and yet we claim them as most excellent antiphlogistics; the former by its producing fatty degeneration of the cellular elements of the inflammatory plasma, reducing it to a milky detritus which is speedily and easily absorbed; the latter, by its antiseptic, antizymotic and antipyretic properties. Quinine arrests putrefaction, it destroys fungi, and, indeed, all low organisms, animal and vegetable; it counteracts the tendency to suppuration, lowers the fever heat, diminishes the oxidation of tissues and the excretion of urea, and, of course, counteracts tissue waste. (See experiments of Harley, Ranke, Kerner, Binz, Calvert, etc.)

Digitalis, tartar emetic, and the refrigerants, saline aperients, bisulphite of soda, the ice-bag and cold water baths, when they are only used at proper stages in appropriate cases, do not act as permanent prostrating remedies, but, on the contrary, often remove pathological conditions which would soon result in irremediable prostration.

On the other hand, will alcoholic stimulants sustain and support the strength of the patient laboring under inflammatory disease; or will they circumscribe or modify the destructive processes and fatal tendencies of inflammation? We do not believe that they have any lengthened strengthening virtues, but are merely artificial, temporary stimuli, increasing for a short time the vital activity, but soon followed by a corresponding depression of power. One of the effects of inflammation is a perversion of the nutrition of tissue; and it is generally agreed amongst physiologists that alcoholic stimulants are not subservient to the nutrition of tissue. One of the effects of inflammation is to increase fever heat, and Professor Parkes and Count Vollonwitz have proved by experiments that these stimulants are not depressors of temperature or of fever heat.

Alcohol when taken into the stomach is quickly absorbed and enters the circulation, from whence a part of it escapes with the secretions and part is probably burnt in the lungs, its carbon uniting with the oxygen of the inhaled air to form carbonic acid, and its hydrogen similarly combined to form water. This combustion in the lungs can scarcely be salutary in the phlegmasiæ; their healthy action will be obstructed, the formation of effete materials increased, and thus a tendency to congestion, exudation, etc.

As to its claims as a nutrient and tonic, Dr. Chambers says: "Alcohol is really the most ungenerous diet there is; it quickens the heart, causes capillary congestion, irregular circulation, and disposes to exudations and engorgements." "Alcohol arrests and obstructs the vigor of vital action; by it growth is checked, as we see by animals kept artificially small by its use when young; and also in men who early in life have indulged in ardent spirits; this very indulgence blunting their appetite for more nutritious substances. . . . . It is very obvious that under its use renewal goes on slower, as we know by the diminished excretion of urea water, bile, etc., and we can hardly, therefore, expect it to be advantageous where the continual renewal of vital power is our primary object." There is no doubt, therefore, that most of the credit given to alcoholic stimulants as supporting remedies is due to the eggs, milk, beef essence and other nutrients which are generally given between the doses of wine, brandy or whisky. It is certainly not good practice or in accordance with sound physiological principles to give these stimulants at the same time as the nutrients, for it has been abundantly proved that alcohol does not aid digestion. It, on the contrary, very materially retards and interferes with it; instead of dissolving the food, it hardens and coagulates the protein compounds in the stomach; precipitates the pepsin so as to render digestion exceedingly difficult. Every physician of large experience, especially in city practice, will often meet with patients who wish for advice for what they term dyspepsia, or perhaps biliousness, or liver complaint; they will tell you they have not the least appetite for breakfast, very little for dinner, but can manage to eat a little at supper, but even that lies heavy on their stomach and does not seem to nourish them; they will also tell you that if they force down a little breakfast they almost invariably vomit it up again. When such patients as these consult me, my first question to them is: Are you in the habit of taking stimulants before your meals? The invariable answer is, "Oh, yes; if I did not take a little whisky or brandy to stimulate my appetite I would be unable to eat a morsel." My advice to them, then, is that if they will certainly abandon the use of all stimulants I will try and relieve them; but if they persist in their use I can promise them no permanent benefit. But Professor Bennett's theory is, that stimulants give tone and power to the weakened action of the heart and to the organic functions generally, and are especially beneficial in pneumonia and other inflammatory diseases, because they assist in carrying forward the natural processes of inflammation. He contends that the inflammatory infiltrate or plastic exudation cannot be got rid of except by suppuration, and as stimulants hasten that change they are, therefore, especially indicated in this class of diseases. If Dr. Bennett is certain of such happy effects from stimulants, why does he not give them more freely, or why should he confess that he thinks that it was overstimulation which caused Dr. Tod to lose one patient in every nine of his pneumonia cases?

The fact is that, although the Edinburgh professor has done more to popularize the stimulating treatment than any other man living, he does not carry out his theory so thoroughly as is done by a great majority of his disciples. In many of his published cases of pneumonia he gives no stimulants at all, but prescribes small doses of tartar emetic or colchicum and spirits of mindererus, acetate of potash, nitre infusion of serpentaria, etc. When he does give stimulants, it is only a few ounces of wine, so that his practice bears no comparison with his theory, whilst his imitators make the practice far outrun the theory; instead of using Dr. Bennett's prescription of small doses of antimony or colchicum and a few ounces of wine, they pour in heroic doses of carbonate of ammonia, and brandy or whisky. Well may Sir William Jenner say that inflammatory diseases now-a-days suffer more from the use of stimulants than from the want of them. We believe with Professor Bennett that venesection, a free use of strong purgatives, calomel, tartar emetic, veratrum viride, etc., would be bad treatment in a great many cases of inflammatory disease; whilst we cordially agree with him in the employment of nutrients, as beef tea, milk, raw or soft-boiled eggs, etc., in a vast majority of cases. We do not recommend bleeding, purging, antimony, mercury, veratrum viride and aconite in a disease because it is a case of pneumonitis, pleuritis, peritonitis or encephalitis, but in certain cases and in certain conditions of these diseases we are firmly persuaded that one or more of these antiphlogistic remedies should be administered; and we most emphatically protest against the indiscriminate and immoderate employment of stimulants which characterizes much of the practice of our modern therapeutic reformers. We heartily approve the Hippocratic maxim, that when we are not certain of doing good we should see to it that we do no harm; but whilst believing and acting up to this doctrine we think we are justified, on the one hand, in condemning the heroic stimulating, and, on the other, the passive or expectant system of treatment. When we are called upon to treat an acute inflammation, we cannot reconcile the lavish administration of stimulants as being in accordance with sound scientific principles; and we are equally decided in our opinion that were we to treat such cases on the expectant plan, with a few poultices and a little liquor ammonia acetates, we would be little better than the homœopaths—trifling with the lives of our patients.

"*Medicus naturæ minister, non magister est,*" is a very popular maxim with those who advocate the expectant system of medication, but they carry out the doctrine beyond its legitimate meaning, for they neither control nor assist nature in her usual processes; as ministers of nature, carefully scanning the way she directs, we should endeavor to aid and assist her in her beneficent and conservative efforts to eliminate, neutralize, modify, circumscribe or subdue disease ; and as nature is sometimes a harsh step-mother, we venture the opinion that we will be occasionally justified in using prompt and active means to control and counteract her course when we distinctly see it tending to destruction and death instead of regeneration and health.

In those cases of acute sthenic pneumonitis in robust subjects, where the pulse is strong and hard, or where the fever heat is over 105°, or where there is threatening cerebral congestion, shall we withhold venesection and thereby hazard the patient's life because many cases of pneumonia have been bled to their hurt? When we see a stout young man struck down with an acute attack of encephalitis, shall we refuse to bleed him because venesection has often been most extravagantly and hurtfully used in cases of apoplexy, ramollissement, or cerebral abscess? I am ever ready to condemn the great use and abuse of mercury under the plea of "torpid liver," "obstructed portal circulation," "surcharge of bile," etc. ; but because a medicine has been indiscriminately used and given in poisonous doses, are we to abandon it altogether? If we can alleviate suffering, and facilitate and hasten the convalescence of a case of pneumonia or pericarditis with a few doses of calomel and opium, are we to ignore the prescription because some cases of these same diseases have been severely salivated by this remedy? In cases of inflammation or fever, where the action of the heart and arteries is rapid and violent, are we to withhold veratrum viride because some cases of the kind have been severely frightened and nauseated with it?

And here I would wish to add my testimony to the safety, efficiency and remedial virtues of this cardiac and arterial sedative. I have used it hundreds of times, and can conscientiously testify that I have never witnessed any serious results from its employment. Even where it is pushed too far and the patient very much nauseated and apparently prostrated with it, a little paregoric and aromatic spirits of ammonia, or some compound spirits of sulphuric ether and compound tincture of cardamon, will soon dispel the alarming symptoms. In the stage of typhus or typhoid fever characterized by boisterous or noisy delirium, wherein most authors recommend alcoholic stimulants, I have seen the most happy effects from the tincture of veratrum and extract of belladonna; three drops of the former and one-quarter of a grain of the latter every three hours until the patient becomes calm and collected. Of course, the veratrum should not be given in the advanced stage of the fever, or when the heart's action is weak. In *post partum* inflammations this remedy is a most valuable antiphlogistic; but in rheumatic, pericardiac or nephritic inflammations the aconite, colchicum or digitalis will be preferable. The antipyretic or refrigerant plan of treating inflammatory diseases is now attracting considerable attention, and as an assistant therapeutic measure with others, it deserves extensive trial. In pleuritis, pneumonitis, encephalitis, acute rheumatism, and in typhus, typhoid, remittent and puerperal fevers, the ice-bags or wet sheets will be found, in many instances, efficient and valuable aids in the treatment.

The internal use of refrigerant medicines, such as acetate, sulphate and nitrate of potassa, and the bisulphite of soda or magnesia, will prove most reliable remedies as antiphlogistics. In *post partum* inflammations, in surgical fever, and, indeed, in most cases where there is any reason to fear septæmia, these sulphites will prove of essential service as antipyretics, refrigerants and antizymotics. One great objection to their free employment is their unpleasant taste and odor, but by adding to their solution a little compound spirit of lavender, essence of peppermint, cinnamon, or any other essence palatable to the patient, they will generally agree quite well without either purging or nauseating.

In concluding our short and imperfect apology for the antiphlogistic treatment, we would again define our position: first, that there are many lamentable evils, morally and medicinally, consequent on the great rebound from the antiphlogistic to the stimulating treatment of disease; second, we firmly believe that the omission and neglect of the antiphlogistic system is fraught with lasting and often imminent danger to the patient; grave and serious structural lesions accruing in numerous instances which might be obviated by prompt and vigorous abortive measures; third, we recognize in the present fashionable sustaining, or restorative, or stimulating plan of treatment, a close assimilation and affinity with homœopathy, whereby the scientific practice of medicine is brought into disrepute and the unfortunate patients permitted to linger for weeks or months from the effects of an acute disease which might have been arrested, or at least circumscribed and controlled, by efficient remedies, so as to promote a speedy and happy convalescence.

